# Immune checkpoint inhibitor-induced bullous pemphigoid: a systematic review of clinical characteristics and outcomes based on case reports

**DOI:** 10.3389/fimmu.2026.1745011

**Published:** 2026-03-04

**Authors:** Lei Chang, Yongjia Cui, Wenping Lu, Siqing Zhao, Zhili Zhuo

**Affiliations:** Department of Oncology, Guang’anmen Hospital, China Academy of Chinese Medical Sciences, Beijing, China

**Keywords:** bullous pemphigoid, case report, immune checkpoint inhibitor, immune-related adverse events, systematic review

## Abstract

**Objective:**

Immune checkpoint inhibitor-induced bullous pemphigoid (ICI-BP) is a rare and complex cutaneous immune-related adverse event (cirAE) that often impacts the continuation of ICI therapy. Currently, there are no prospective clinical studies addressing the optimal management of BP alongside ICI continuation, with existing evidence largely derived from case reports or series. This study systematically analyzes published case reports and series to compile evidence regarding the management of ICI-BP and ICI rechallenge, aiming to inform clinical practice.

**Methods:**

A comprehensive search of the PubMed, Embase, and Web of Science Core Collection (WoS CC) databases was conducted from their inception to identify eligible case reports and series. Relevant data were extracted using a standardized form. A total of 116 cases from 89 publications were included in the analysis.

**Results:**

There was no discernible disparity in the final response rate between patients with mild and severe BP (p > 0.05); however, the percentage of severe patients undergoing escalation therapy was notably higher (p < 0.001). This suggests that employing an active treatment approach based on disease severity effectively ameliorated the potentially graver prognosis in severe cases. Following BP management, 18 patients underwent rechallenge with ICI therapy, with an overall low BP recurrence rate (22.2%), indicating that most patients tolerated the resumption well. Nevertheless, the majority of relapses transpired in patients with initial severe BP, with mucosal invasion, and those who restarted the original therapy, pointing to an elevated risk in this cohort. The 11 deceased patients constituted a high-risk subset distinguished by advanced age (median 74 years), a prevalence of melanoma, and severe BP. The duration from BP diagnosis to demise was briefer in this subset (median 3.7 months), with half of the patients succumbing within 4 months.

**Conclusion:**

ICI-BP is usually manageable through a stepwise therapeutic approach, and resuming ICI treatment after lesion remission is generally possible. Clinical decision-making must be tailored to the individual patient. Caution and careful monitoring are essential when reinitiating ICI in patients who initially presented with severe disease or mucosal involvement. For elderly patients, those with melanoma, and individuals experiencing severe BP, heightened vigilance is necessary concerning short-term prognostic risks.

**Systematic review registration:**

https://www.crd.york.ac.uk/prospero/, identifier CRD420251172315

## 1 Introduction

Immune checkpoint inhibitors (ICIs) have transformed the treatment landscape for advanced cancers, holding the potential for prolonged survival in patients with previously dismal prognoses (1–3). While ICIs work by reigniting anti-tumor immune responses through lifting inhibitory signals on T cells, they can also trigger immune-related adverse events (irAEs) by inciting attacks on healthy tissues (4). Among the various irAEs, cutaneous toxicities are prevalent, with bullous pemphigoid (BP) standing out as a rare yet potentially severe skin reaction that is gaining prominence in clinical practice (5).

Immune checkpoint inhibitor-induced bullous pemphigoid (ICI-BP) demonstrates high variability in clinical presentation, onset time, and severity despite its low overall incidence (approximately 0.3%–1%) (6, 7). The spectrum of manifestations ranges from mild pruritus and localized blisters to rapidly advancing lesions affecting a large body surface area (8). This variability not only significantly diminishes patients’ quality of life but also frequently poses a crucial dilemma for clinicians: whether to halt potentially effective anti-cancer treatment. The scarcity of ICI-BP cases hampers the availability of robust clinical evidence, leading to a reliance on case reports and series, with a lack of prospective clinical trials impeding the development of optimal management approaches.

Previous systematic reviews have offered valuable insights into the clinical characteristics of ICI-BP (9); however, several fundamental questions essential for clinical decision-making remain insufficiently addressed. For example, how does the discontinuation of ICI therapy and the subsequent decision regarding rechallenge affect patient oncological outcomes? Which treatment strategies are most effective for severe or refractory cases? Furthermore, does the severity of BP influence tumor outcomes? Addressing these questions is crucial for achieving an optimal balance between managing toxicity and maintaining anti-tumor efficacy.

This systematic review seeks to enhance current literature by conducting a thorough analysis of published case reports. It will address two primary challenges in managing ICI-BP. First, it will conduct a detailed examination of modifications in ICI treatment, such as holds and rechallenges, and their impact on patient tumor response. Second, it will summarize treatment options for severe or refractory ICI-BP systematically, offering more precise references for clinical management. By presenting this updated evidence synthesis focusing on critical clinical decision points, we aim to provide practical guidance for oncologists and dermatologists to optimize the management of ICI-BP patients.

## 2 Materials and methods

### 2.1 Search strategy

**Figure 1:**
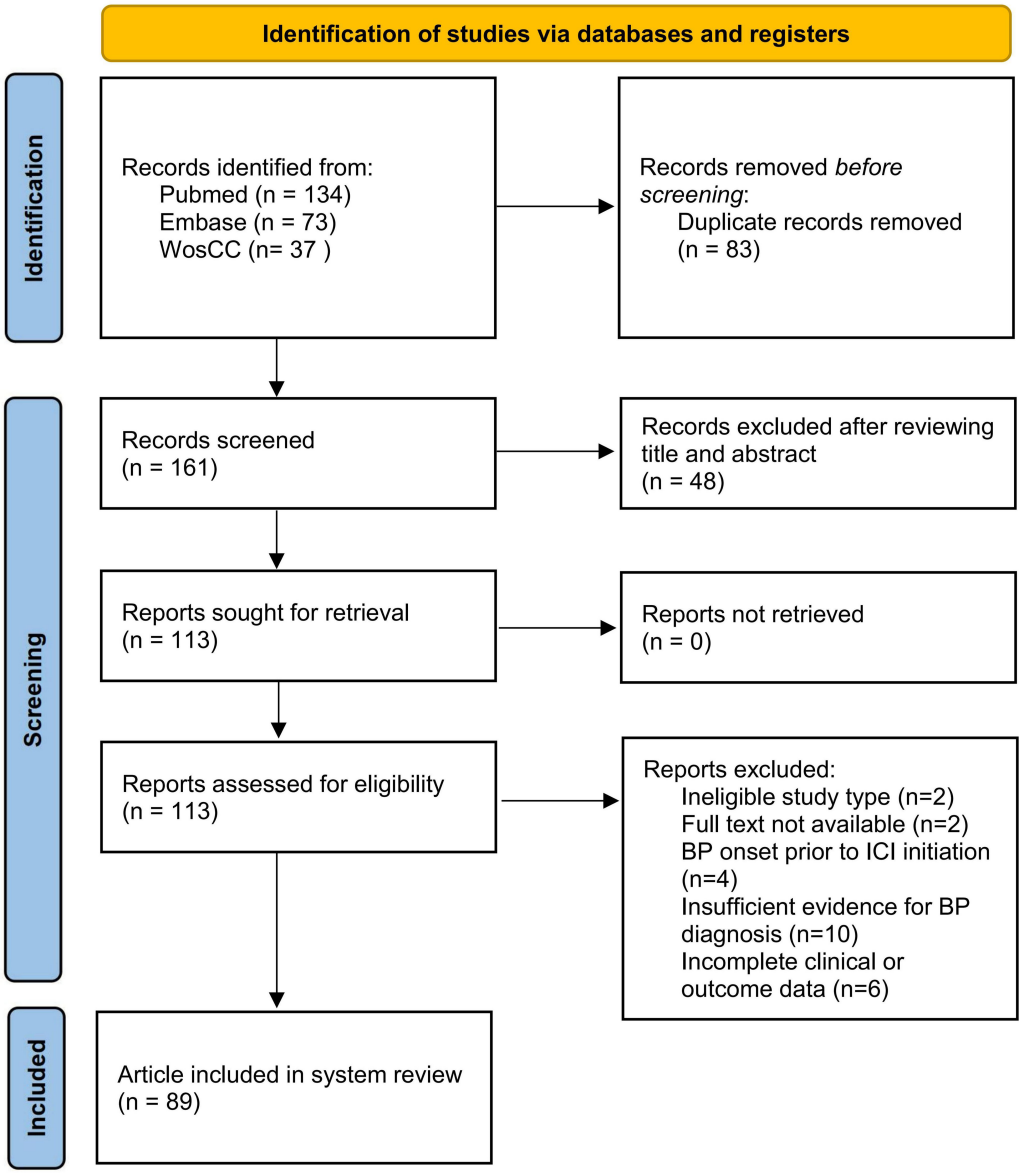
Data collection and retrieval strategy.

A thorough search was performed on various databases such as PubMed, Embase, and Web of Science Core Collection (WoS CC) using subject terms and keywords pertaining to neoplasms, immune checkpoint inhibitors, bullous pemphigoid, and case reports. The date for the literature search was October 20, 2025. The objective was to locate all pertinent English-language articles published since the establishment of each database. The specific search strategy can be found in the **Supplementary Materials**. This study has been registered with International Prospective Register of Systematic Reviews (PROSPERO) under registration number CRD420251172315. The detailed study flow diagram is presented in **Figure 1**.

### 2.2 Eligibility criteria

EndNote was utilized for literature management. Following the deduplication of records from three databases, two investigators (CL and CYJ) independently screened titles and abstracts and performed quality assessments of the case reports. Inclusion criteria were as follows: research on tumor-associated ICI-BP must include published articles with full-text availability, specifically focusing on case reports or case series. The diagnosis must satisfy all of the following criteria. 1) Clinical evidence: The presence of rashes must be consistent with BP characteristics, such as pruritic wheals, vesicles, and bullae. 2) Histopathological evidence: Biopsy results must demonstrate subepidermal fissures, typically accompanied by eosinophilic infiltration. 3) Immunopathological evidence requires at least one of the following criteria: a) direct immunofluorescence must show linear deposition of IgG and/or C3 along the basement membrane. b) Indirect immunofluorescence must detect anti-basement membrane band antibodies in serum. c) Serological testing must identify circulating autoantibodies against BP180 and/or BP230 using ELISA or immunoblotting. Exclusion criteria included reviews, systematic reviews, books, editorials, or conference abstracts, cases with concurrent inflammatory or blistering dermatoses that could confound BP diagnosis (e.g., lichen planus, Grover’s disease, or psoriasis), BP onset before ICI therapy, and insufficient key information like tumor type or ICI agent used.

### 2.3 Study selection and quality assessment

The study selection was independently and redundantly conducted by three reviewers (CL, CYJ, and ZSQ). The evaluation criteria comprised the following: 1) demographic information, 2) a documented history of ICI use, 3) a precise timeline detailing the onset of BP symptoms and diagnosis, 4) BP diagnosis confirmed through direct immunofluorescence (DIF) and/or serological methods, 5) a comprehensive description of the intervention or treatment outcomes, 6) a thorough account of clinical status following the intervention, 7) identification and description of adverse reactions (harm) or unforeseen events, and 8) a clear presentation of results. Studies that fulfilled all criteria were included in the analysis. In cases of disagreement, the third evaluator rendered the final decision. A detailed flowchart of the study is presented in **Figure 1**.

### 2.4 Data extraction

Three investigators (CL, CYJ, and ZSQ) independently reviewed the full texts of all included studies and performed data extraction using a standardized form. This process was followed by a cross-verification step to ensure accuracy. The extracted data encompassed the following domains: publication details (title, first author, and publication year), patient demographics and baseline characteristics (sex, age, past medical history, cancer type, and cancer stage), ICI treatment information (ICI type, specific agent, line of therapy, start date, concomitant anti-tumor therapies, and prior ICI exposure including types and agents), characteristics of ICI-BP occurrence (time of initial skin symptoms, description of presentation, time of BP diagnosis, mucosal involvement, disease severity, and time from ICI initiation to BP onset), management of ICI-BP (initial treatment, initial treatment response, need for treatment escalation, all BP-related treatments received, effective treatment modalities, and time from BP diagnosis to disease control), and other relevant outcomes (ICI discontinuation status at BP onset, impact of BP on ICI therapy, tumor status during BP treatment and at final follow-up, ICI rechallenge attempts, ICI-BP recurrence, and other concurrent immune-related adverse events). All extracted data were compiled and managed in a centralized online Excel database accessible to all co-authors for review and reference.

### 2.5 Data analysis

Descriptive statistics summarized the demographic and clinical characteristics of the cases. Quantitative variables were reported as median and range (minimum to maximum values), and categorical variables were presented as counts and percentages. The Mann–Whitney U test was employed to compare continuous variables across groups, while the chi-square test or Fisher’s exact test was utilized for comparing categorical variables between groups. All analyses were conducted using the SPSS 26.0 software, and a p-value <0.05 was considered statistically significant.

## 3 Result

The initial database search yielded 161 articles. Among these, 48 articles (29.8%) were excluded based on title and abstract screening. Out of the 113 articles subjected to full-text review, 24 articles (21.2%) were excluded for various reasons: two articles (1.8%) were excluded due to unavailability of full-text downloads, two articles (1.8%) lacked case report content, four articles (3.5%) reported BP occurrences before ICI use, another four articles (3.5%) did not align with BP diagnosis criteria, six articles (5.3%) had incomplete medical history or treatment information, and six full-text articles along with five individual cases from other studies lacked histopathological or immunopathological evidence. Following screening, 116 cases from 89 articles met the inclusion criteria and were analyzed in the systematic review’s methodology section.

### 3.1 Patient baseline characteristics

**Table 1** presents the key demographics of the participants. The majority was male (87/116, 75.00%), with a median age of 71.5 years (range, 24–90). Individuals aged 60 years or older made up 87.93% of the group. The most common primary tumor types were melanoma (41.38%), lung cancer (21.56%), renal cell carcinoma (14.66%), and urothelial cancer (4.31%).

**Table 1:** Characteristics of patients with ICI-BP.

N = 116	n	Proportion
Gender		
Male	87	75.00%
Female	29	25.00%
Primary cancer		
Melanoma	48	41.38%
Lung cancer	25	21.56%
Renal cell carcinoma	17	14.66%
Urothelial cancer	5	4.31%
Head and neck squamous cell carcinoma	3	2.59%
Cutaneous squamous cell carcinoma	3	2.59%
Endometrial cancer	2	1.72%
Colorectal cancer	2	1.72%
Hepatocellular carcinoma	2	1.72%
Other*	9	7.76%
NA	1	0.77%
ICI type		
Anti-PD-1	98	84.48%
Anti-PD-1 and anti-CTLA-4	7	6.03%
Anti-PD-L1	5	4.31%
Anti-CTLA-4	2	1.72%
Anti-PD-L1 and anti-CTLA-4	1	0.86%
Anti-PD-1 and anti-LAG3	1	0.86%
Anti-PD-1 and anti-ILT4	1	0.86%
Anti-PD-1/TGFβ	1	0.86%
ICI agents		
Nivolumab	49	42.24%
Pembrolizumab	41	35.34%
Ipilimumab + nivolumab	7	6.03%
Atezolizumab	4	3.45%
Cemiplimab	3	2.59%
Sintilimab	3	2.59%
Ipilimumab	2	1.72%
Durvalumab + tremelimumab	1	0.86%
Nivolumab + relatlimab	1	0.86%
Pembrolizumab + MK4830	1	0.86%
Bintrafusp alfa	1	0.86%
ICI treatment line		
0	7	6.03%
1	50	43.10%
2	17	14.66%
≥3	8	6.90%
NA	34	29.31%
Prior ICI agents		
Ipilimumab	7	46.67%
pembrolizumab	3	20.00%
Nivolumab	2	13.33%
Ipilimumab + nivolumab	2	13.33%
Ipilimumab + pembrolizumab	1	6.67%
ICI treatment already completed at the onset		
Yes	16	13.79%
No	95	81.90%
NA	5	4.31%
Concomitant tumor therapies at BP onset		
Targeted therapy	7	6.03%
Chemotherapy	6	5.17%
Radiotherapy	6	5.17%
Targeted therapy, hormonal therapy	1	0.86%
None	96	82.76%%
BP-related symptoms		
Pruritus (itching)	78	67.24%
Erythema/rash	93	80.17%
BP diagnosis method		
Histopathologic examination (HE)	116	100%
DIF	107	92.24%
IIF	22	18.97%
BP180	62	53.44%
BP230	12	10.34%
CTCAE grade of BP		
1	1	0.86%
2	54	46.55%
3	50	43.10%
4	2	1.72%
NA	9	7.76%
BP treatment		
Systemic CS	97	83.62%
Topical CS	86	74.14%
Tetracycline antibiotics	36	31.03%
Biological treatments	30	25.86%
Immunosuppressants	19	16.40%
nicotinamide	18	15.51%
Dapsone	12	10.34%
Antihistamines	11	9.48%
IVIG	6	5.17%
Plasma exchange	2	1.72%
NA	13	11.21%
Treatment escalation		
Yes	63	54.31%
No	51	43.97%
NA	2	1.72%
IBP outcome		
CR	57	49.14%
PR	50	25.86%
No Response (NR)	5	4.31%
NA	4	3.45%
Management of ICI		
Permanently discontinued	16	13.79%
Continued without interruption	20	17.24%
Continued until tumor response	3	2.59%
Interrupted and later resumed	18	15.52%
Interrupted and not resumed	54	4.66%
NA	5	4.31%
Tumor outcome		
CR	14	12.07%
PR	5	4.31%
SD	18	15.52%
PD	7	6.03%
Death	11	9.48%
NA	60	51.72%

ICI-BP, immune checkpoint inhibitor-induced bullous pemphigoid; CS, corticosteroid therapy; DIF, direct immunofluorescence; IIF, indirect immunofluorescence; IVIG, intravenous immunoglobulin; CR, complete response; PR, partial response; PD, progressive disease.

^*^Other (cervical cancer 1, gastric cancer 1, Hodgkin lymphoma 1, head and neck squamous cell carcinoma and lung cancer 1, invasive ductal carcinoma and soft tissue sarcoma 1, penile squamous cell carcinoma 1, earlobe squamous cell carcinoma 1, oropharyngeal cancer 1, and gastric cancer 1).

The statistical analysis of treatment lines indicated that first-line therapy was the most prevalent, accounting for 50 cases (43.10%), followed by second-line therapy with 17 cases (14.66%), third-line and above with eight cases (6.90%), second-line therapy again with 13 cases (10.0%), and adjuvant therapy with seven cases (6.03%).

The most frequently utilized ICI type was anti-PD-1, representing 84.48% (98/116), followed by combination therapies in 10 cases (8.62%), which included anti-PD-1 and anti-CTLA-4 in seven cases (10–16), anti-PD-L1 and anti-CTLA-4 in one case (17), anti-PD-1 and anti-LAG3 in one case (14), and anti-PD-1 and anti-ILT4 in one case (18). Other ICI types included anti-PD-L1 in five cases (4.31%) (19–23), anti-CTLA-4 in two cases (1.72%) (24, 25), and anti-PD-1/TGF-β in one case (26). The ICI regimen comprised nivolumab in 49 cases (42.24%), pembrolizumab in 41 cases (35.34%), ipilimumab plus nivolumab in seven cases (6.03%), atezolizumab in four cases (3.45%), cemiplimab in three cases (2.59%), sintilimab in three cases (2.59%), ipilimumab in two cases (1.72%), and durvalumab combined with tremelimumab, nivolumab with relatlimab, pembrolizumab with MK4830, bintrafusp alfa, karelizumab, durvalumab, and tislelizumab in one case each (0.86%).

ICI therapy history was present in 12.93% of cases, with prior use of ipilimumab in seven cases (23, 27–31), pembrolizumab in three cases (24, 26, 32), nivolumab in two cases (25, 33), a combination of ipilimumab and nivolumab in two cases (10, 34, 35), and a combination of ipilimumab and pembrolizumab in one case.

Of the total cases, 13.79% (16/116) had finished ICI treatment when diagnosed with BP. Concurrent use of other tumor therapies represented 17.24% of cases, with 6.03% combined with targeted therapy (12, 16, 36–40), 5.17% with radiotherapy (32, 35, 41–44) or chemotherapy (16, 45–49), and 0.86% with a combination of targeted therapy and endocrine therapy (16). The most common pre-existing medical conditions were hypertension (12.93%) and type 2 diabetes (8.62%). A history of dermatological conditions was documented in 3.45% of cases, including psoriasis in two cases and Grover’s disease in two cases. Additionally, three cases had previous autoimmune diseases, such as Crohn’s disease, autoimmune hypophysitis, and autoimmune thyroid disease.

### 3.2 ICI-BP symptoms and time of onset

Pruritus occurred in 67.24% of patients, erythema or rash in 80.17%, and mucosal involvement in 19.83% (50). The severity of BP was categorized according to Common Terminology Criteria for Adverse Events (CTCAE) 5.0, with mild cases (grades 1–2) accounting for 47.41% and moderate-to-severe cases (grades 3–4) for 44.83%.

An analysis of data from 83 cases showed that the median onset time of ICI-BP-related skin symptoms was 21.4 weeks (range, 0–283 weeks), with an average of 41.21 weeks (potentially skewed rightward due to some larger values inflating the mean). Some symptoms manifested within 1 week of ICI initiation, while others appeared more than 36 months after treatment. Nevertheless, diagnosis often faced delays, with a median delay of 4.65 weeks (range, 0–115.7 weeks) and an average delay of 12.38 weeks.

In addition to BP, 26 cases reported other concurrent adverse reactions, including dysthyroidism (3.45%), vitiligo (3.45%), and diarrhea (3.45%), which are common immunotherapy-related adverse effects.

### 3.3 BP diagnosis

Histopathological evidence of BP was a prerequisite for inclusion. Moreover, 92.23% of cases were diagnosed via DIF, while 18.97% relied on indirect immunofluorescence (IIF) for diagnosis. Among these cases, 53.45% tested positive for BP180 and 10.34% for BP230.

### 3.4 BP management

In the selection of treatment regimens, 94.83% of patients were administered corticosteroids, in either systemic or topical forms. Among these patients, 83.62% received systemic corticosteroid therapy (CS), while 74.14% were treated with topical CS. Additionally, 31.03% were prescribed tetracycline antibiotics (41, 51, 52) and is often used in combination with nicotinamide (53, 54), and 25.86% received biological therapies, which included omalizumab (4) (47, 55–57), rituximab (11) (42, 58–60), dupilumab (17) (61–63), and infliximab (1) (43). Furthermore, 16.38% of patients were given immunosuppressants (64, 65), 15.52% received nicotinamide, 10.34% were treated with dapsone (13, 15, 27, 34, 47, 66–71), 9.48% received antihistamines (72, 73), 5.17% were administered intravenous immunoglobulin (IVIG) (46, 47, 69, 74–76), and 17.24% underwent plasma exchange (55, 77).

Of the patients, 54.31% experienced treatment escalation. We recorded the efficacy of all medications administered throughout the treatment course. Oral glucocorticoids constituted the fundamental basis of therapy and were the most frequently reported effective treatment regimen (n = 82). Topical potent or ultrapotent glucocorticoids (n = 36) were often used in conjunction with them for primary lesion management. When reducing glucocorticoid dosage or as an alternative therapy, tetracyclines (doxycycline/minocycline, n = 20) combined with nicotinamide (n = 8) emerged as the most commonly adopted regimens, reflecting a preference for non-steroidal immunomodulatory strategies. Traditional methotrexate (n = 11) also served as a common agent for glucocorticoid tapering. The treatment escalation pathway was evident in moderate-to-severe, refractory, or glucocorticoid-dependent cases. Dupilumab (n = 16) was the most frequently reported biologic agent, highlighting its central role in the current management of refractory ICI-BP. Rituximab (n = 9) was utilized for more severe or treatment-resistant cases. A minority of critical cases received intravenous immunoglobulin (n = 4) or plasma exchange (n = 2). Notably, intravenous methylprednisolone (n = 9) was administered to some patients requiring rapid disease control. Other immunosuppressants, such as mycophenolate mofetil and cyclosporine, along with omalizumab, were also reported sporadically.

Following treatment with ICI-BP, 49.14% of patients achieved a complete response (CR) (78–81), while 43.10% attained a partial response (PR) (82, 83), resulting in a disease control rate of 92.24%. The median duration from the initiation of treatment to disease control was 6 weeks (range, 1–167 weeks), with a mean duration of 12.57 weeks.

### 3.5 Impact of BP on ICI therapy

At the time of the occurrence of BP, 13.79% of patients had completed ICI treatment. Among those affected by BP, 17.24% continued ICI without interruption, 2.59% maintained ICI until achieving tumor response, 15.52% interrupted treatment and later resumed, and 45.56% interrupted and did not resume ICI. Eighteen patients restarted ICI treatment after experiencing BP, and seven of these patients subsequently experienced a recurrence of BP following the ICI restart.

### 3.6 Oncologic outcomes

We conducted a statistical analysis of tumor progression throughout the follow-up period. Among the 55 cases with reported tumor outcomes, the disease control rate was 67.27%, comprising 14 cases of CR (84–87), five cases of PR (88), and 18 cases of stable disease (SD) (89, 90). Additionally, there were seven cases of progressive disease (PD) and 11 cases resulting in death.

### 3.7 Stratified analysis

#### 3.7.1 Comparison of BP onset characteristics and outcomes between mild-to-moderate and severe patients

Stratified analysis according to severity indicated that patients with grade 1–2 compared to those with grade 3–4 BP exhibited no differences in BP outcomes, tumor outcomes, or ICI type, with the exception of a significant difference regarding the decision to escalate BP treatment (p < 0.001). Related results are shown in **Table 2**.

**Table 2:** Comparison of BP onset characteristics and outcomes between mild-to-moderate and severe patients.

Characteristics	CTCAE 1–2	CTCAE 3–4	Test statistic	p
Gender (M/F)	42/13	38/14	χ^2^ = 0.153	0.696
Age	70 (31–90)	70.5 (24–85)	U = 1264.5	0.302
ICI type (anti-PD-1/anti-PD-L1/anti-CTLA-4/combined)	46/3/1/5	44/2/1/5	χ^2^ = 0.160	0.984
Prior ICI (yes/no)	8/47	7/45	χ^2^ = 0.026	0.872
Time from ICI to cutaneous symptom onset (weeks)	26 (1–282.9)	17.55 (0–158.6)	U = 697.0	0.142
Diagnosis delay time (weeks)	5.3 (0–60)	4.65 (0–115.7)	U = 754.0	0.710
Mucosal involvement (yes/no)	11/35	12/32	χ^2^ = 0.133	0.715
BP outcome (CR/PR/NR)	26/24/2	24/24/3	χ^2^ = 0.270	0.874
Treatment escalation (yes/no)	18/36	36/15	χ^2^ = 14.573	0.000
Tumor outcome (CR/PR/SD/PD/death)	5/4/11/3/2	8/1/6/3/8	χ^2^ = 7.771	0.169

BP, bullous pemphigoid; ICI, immune checkpoint inhibitor; CR, complete response; PR, partial response; PD, progressive disease.

#### 3.7.2 Profile of patients undergoing ICI rechallenge

This study investigated 18 patients who underwent rechallenge with ICI after a hiatus due to BP concerns. Patient information regarding the resumption of treatment is presented in **Table 3**. Among these patients, the most prevalent primary tumors were melanoma (six cases, 33.3%) and lung cancer (four cases, 22.2%). The initial ICI regimens that triggered BP consisted mainly of anti-PD-1/PD-L1 monotherapy (15 cases) or their combination with anti-CTLA-4 agents (three cases). The majority of BP cases were classified as grade 2 (10 cases, 55.6%) or grade 3 (eight cases, 44.4%) in terms of severity according to the CTCAE grading system. Importantly, in the documented cases, most patients did not exhibit mucosal involvement (10 cases definitively “no” and only four “yes”). Thirteen patients (72.2%) resumed the same drugs that initially led to BP (original drug resumption), while the remaining five switched to alternative medications. BP recurrence occurred in four cases (22.22%) after resuming ICI treatment, with three cases initially graded as 3 and one case as 2, all of whom restarted the original medications, and two of whom had mucosal involvement. In the context of ICI-associated BP, restarting ICI therapy (especially the original regimen) post-successful skin lesion management appears to be a clinically viable choice for most patients, with an overall low BP recurrence rate (22.2%). Nevertheless, a more severe initial presentation (grade 3), mucosal involvement, and reinstatement of the original drug may be linked to a heightened risk of recurrence, necessitating vigilant monitoring.

**Table 3:** Profile and outcomes of the ICI rechallenge cohort (n = 18).

Rank	Gender	Age	Tumor type	ICI type	ICI target	Mucosal involvement	Disease severity of BP	BP outcome	Tumor outcome	Rechallenge ICI agents	BP recurrence in ICI rechallenge	Ref.
1	Female	77	Lung cancer	Anti-PD-1	Nivolumab	No	3	PR	NA	NA	NA	(57)
2	Male	72	Melanoma	Anti-PD-1	Pembrolizumab	Yes	3	PR	Death	Ipilimumab	NA	(91)
3	Male	63	Head and neck squamous cell carcinoma	Anti-PD-1	Nivolumab	Yes	3	CR	PD	Nivolumab	Yes	(12)
4	Male	64	Melanoma	Anti-PD-1	Pembrolizumab	No	2	CR	NA	Pembrolizumab	Yes	(92)
5	Female	65	Lung cancer	Anti-PD-L1 and anti-CTLA-4	Durvalumab + tremelimumab	No	2	CR	NA	NA	NA	(17)
6	Male	87	Urothelial cancer	Anti-PD-L1	Atezolizumab	No	2	CR	NA	Atezolizumab	No	(20)
7	Male	62	Renal cell carcinoma	Anti-PD-1	Nivolumab	No	2	CR	NA	Nivolumab	No	(33)
8	Female	76	Melanoma	Anti-PD-1 and anti-CTLA-4	Nivolumab + ipilimumab	No	2	CR	NA	NA	NA	(14)
9	Female	77	Lung cancer	Anti-PD-1	Pembrolizumab	Yes	2	PR	SD	Pembrolizumab	Yes	(93)
10	Male	67	Renal cell carcinoma	Anti-PD-1	Nivolumab	NA	2	CR	SD	Nivolumab	No	(44)
11	Male	59	Colorectal cancer	Anti-PD-1/TGF-β	Bintrafusp alfa	No	3	CR	NA	Nivolumab + ipilimumab	No	(26)
12	Male	78	Cutaneous squamous cell carcinoma (CSCC)	Anti-PD-1	Cemiplimab	NA	2	PR	NA	Cemiplimab	No	(94)
13	Male	73	Lung cancer	Anti-PD-1	Pembrolizumab	NA	3	PR	PD	Pembrolizumab	No	(75)
14	Male	80	Metastatic cutaneous squamous cell carcinoma	Anti-PD-1	Cemiplimab	NA	2	NA	NA	Cemiplimab	No	(95)
15	Male	67	Melanoma	Anti-PD-1	Pembrolizumab	Yes	2	PR	CR	Pembrolizumab	Yes	(56)
16	Female	70	Endometrial cancer	Anti-PD-1	Pembrolizumab	No	3	PR	Death	Pembrolizumab	No	(39)
17	Male	61	Oropharyngeal squamous cell carcinoma	Anti-PD-1	Nivolumab	No	3	PR	NA	Nivolumab	No	(96)
18	Female	62	Melanoma	Anti-PD-1 and anti-CTLA-4	Ipilimumab and nivolumab	No	3	CR	CR	Nivolumab	No	(11)

ICI, immune checkpoint inhibitor; BP, bullous pemphigoid; CR, complete response; PR, partial response; PD, progressive disease.

When evaluating the impact of treatment strategies on tumor prognosis, we found no statistically significant association between restarting ICI and tumor outcomes (**Table 4**).

**Table 4:** Tumor progression in relation to ICI management strategies and BP treatment outcomes.

Characteristics	Tumor outcome (CR/PR/SD/PD/death)
ICI rechallenge	2/0/2/2/2
ICI permanent discontinue	7/1/11/2/6
χ^2^	−0.55
p	0.58
ICI continue	1/1/2/2/2
ICI discontinue	11/4/16/5/9
χ^2^	−0.808
p	0.420

ICI, immune checkpoint inhibitor; BP, bullous pemphigoid; CR, complete response; PR, partial response; PD, progressive disease.

#### 3.7.3 Profile of deceased patients

Among the 11 patients who ultimately succumbed, their information is detailed in **Table 5**. The analysis indicates a predominance of male patients, comprising eight cases (72.7%), with a median age of 74 years (range, 68–82 years). Melanoma was the most prevalent tumor type (five cases, 45.5%), followed by head and neck squamous cell carcinoma (two cases), lung cancer (two cases), urothelial carcinoma, and endometrial carcinoma (one case each). All patients were treated with anti-PD-1 monotherapy (90.91%) or a combination of anti-PD-1 and anti-ILT4 (0.91%). Two patients had prior exposure to ICI therapy (both with ipilimumab). The majority of cases (eight cases) were classified as stage 3 BP, while the rest were stage 2 (one case), and one case had an unknown stage. The vast majority of patients (10 cases, 90.91%) achieved disease control (CR or PR) through treatment. The median duration from BP diagnosis to death was 3.7 months (range, 2–16 months) among the six cases with available data, with half of the patients (3/6) passing away within 4 months of diagnosis, indicating a poor short-term prognosis associated with BP onset in this cohort. This outcome could be linked to the critical nature of BP, advanced age, treatment-related complications, or rapid tumor progression. Notably, one lung cancer patient had the longest interval from BP diagnosis to death (16 months), while one melanoma patient had the shortest (2 months).

**Table 5:** Profile of non-survivors.

Rank	Gender	Age	Tumor type	ICI type	ICI target	Prior ICI agents	Disease severity of BP	BP outcome	BP diagnosis to death time	Ref.
1	Male	75	Melanoma	Anti-PD-1	Pembrolizumab	Ipilimumab	3	PR	2 months	(27)
2	Male	72	Melanoma	Anti-PD-1	Pembrolizumab	None	3	PR	NA	(91)
3	Male	68	Melanoma	Anti-PD-1	Pembrolizumab	None	3	PR	NA	(12)
4	Male	82	Melanoma	Anti-PD-1	Pembrolizumab	None	3	NR	4 months	(40)
5	Female	80	Lung cancer	Anti-PD-1	Nivolumab	None	3	CR	16 months	(97)
6	Male	70	Melanoma	Anti-PD-1	Pembrolizumab	Ipilimumab	2	CR	10 months	(31)
7	Male	81	Urothelial cancer	Anti-PD-1	Pembrolizumab	None	3	PR	NA	(98)
8	Male	69	Lung cancer	Anti-PD-1	Pembrolizumab	None	3	PR	3.4 months	(46)
9	Female	70	Endometrial cancer	Anti-PD-1	Pembrolizumab	None	3	PR	NA	(39)
10	Female	74	Head and neck squamous cell carcinoma	Anti-PD-1 and anti-ILT4	Pembrolizumab + MK4830	None	2	PR	3.3 months	(18)
11	Male	74	Squamous cell carcinoma of head and neck	Anti-PD-1	Pembrolizumab	None	NA	PR	NA	(16)

ICI, immune checkpoint inhibitor; BP, bullous pemphigoid; CR, complete response; PR, partial response.

In instances where the cause of death was explicitly mentioned, one patient succumbed to pneumonitis, with potential involvement of methotrexate administered for BP treatment (91); another patient’s demise was attributed to cancer (97); a third patient expired from neurological complications without compelling indications of cancer advancement (31); a fourth patient’s death resulted from gastrointestinal bleeding (46); and a fifth patient’s demise was due to metastatic cancer (18).

## 4 Discussion

This article analyzed 116 cases of ICI-BP, demonstrating that ICI-BP can generally be managed through stepwise therapy, and resuming ICI treatment after lesion regression is typically feasible. Clinical decision-making should be personalized: cautious monitoring is necessary when considering ICI re-administration in patients with severe initial symptoms or mucosal involvement, while careful attention is warranted for short-term prognosis risks in elderly individuals, melanoma patients, and those with severe BP. The study utilized stringent inclusion criteria and meticulously planned methodological approaches to offer in-depth insights into the clinical features and treatment of ICI-associated BP. In contrast to previous systematic reviews (9), which aimed for a comprehensive documentation of the disease profile by encompassing all available cases, our study applied stricter inclusion and quality evaluation standards, explicitly excluding cases lacking reported histopathological or immunopathological findings. This methodology ensured that our study group represented a well-defined “typical” ICI-BP population, striving for less biased and more credible conclusions on critical clinical aspects. Moreover, all included case reports underwent standardized quality assessments. This proactive evaluation of raw data quality bolstered the overall reliability and quality of our final dataset. It is through this refined and high-quality dataset that we identified associations not previously addressed in earlier studies, seeking to furnish evidence for the management and resumption of ICI therapy in ICI-BP.

### 4.1 Clinical spectrum and natural history of ICI-BP

Our study cohort validates common features of ICI-BP patients, including a prevalence among elderly male patients, often associated with melanoma and lung cancer, primarily induced by anti-PD-1 agents. This demographic and cancer type distribution closely mirrors actual ICI usage trends and findings from an earlier analysis of the FDA Adverse Event Reporting System (FAERS) database (99). Notably, while the majority of incidents occurred during initial treatment, occurrences were spread throughout all stages of therapy, highlighting the potential for BP to emerge as a side effect at any phase of ICI treatment, underscoring the need for ongoing vigilance among healthcare providers.

Regarding the temporal relationship between ICI therapy and BP onset, our findings reveal a median time of 21.4 weeks post-ICI initiation for BP onset, with a broad range from 0 to 283 weeks. This variability highlights the occurrence of ICI-BP as either an “early” event, sometimes within 1 week of drug administration, or a “delayed” immune-related adverse event. Additionally, 13.85% of patients received a BP diagnosis only after completing ICI therapy, indicating persistent and potentially irreversible immune system overactivation. This emphasizes the crucial need to investigate prior medication history before treatment initiation and to conduct prolonged post-discontinuation surveillance.

A critical discovery is the notable and common delay in diagnosing ICI-BP, with a median of 4.7 weeks and a mean of 12.4 weeks. This timeframe is briefer than the usual diagnostic delay observed in classic BP (100), possibly due to the vigilant monitoring of patients on ICIs, leading to prompt attention to any related skin manifestations. Conversely, extended diagnostic delays over 4 months in classic BP frequently pertain to lesions localized to a single body area. This discovery conveys a significant clinical alert: in patients undergoing ICI treatment, especially elderly men, the presence of persistent, treatment-resistant itching or unusual skin rash should strongly indicate prodromal BP, necessitating prompt investigations like skin biopsy for a conclusive diagnosis. The average diagnostic delay of 94.74 days necessitates reduction, as minimizing this timeframe is essential for promptly commencing tailored therapy, enhancing patient well-being, and potentially impacting prognosis.

Additionally, 44.83% of cases in this cohort were classified as CTCAE grades 3–4, with 19.83% exhibiting mucosal involvement, underscoring that ICI-BP is frequently a moderate-to-severe condition necessitating systemic immunosuppressive treatment. Hypertension and diabetes were the prevailing pre-existing comorbidities (71, 95). Chronic metabolic diseases often coincide with chronic, low-grade inflammatory conditions, potentially impacting immune system homeostasis (101, 102). This interplay between metabolic inflammation and autoimmune responses triggered by ICI may influence BP susceptibility or severity, necessitating further mechanistic exploration. Although the prevalence of patients with documented dermatological conditions (7.76%, mainly psoriasis) and autoimmune diseases (2.59%) in this cohort was modest, their presence carries significant clinical implications. These patients already demonstrate immune dysregulation (103–105), and ICI treatment could exacerbate immune tolerance disruption, leading to new autoimmune manifestations like BP. This underscores the critical importance of conducting a comprehensive medical history review (especially concerning dermatological and autoimmune disorders) before commencing ICI therapy to identify high-risk groups and facilitate enhanced monitoring.

### 4.2 Stepped-care treatment model and challenges in ICI-BP

This study offers a comprehensive analysis of the treatment patterns, clinical progression, and influence on oncologic results of ICI-BP, uncovering intricate management approaches and eventual outcomes of this immune-related adverse event.

#### 4.2.1 Stepped and individualized treatment pathway

Our data clearly delineate a standardized treatment approach for ICI-BP. The primary treatment revolves around corticosteroids (94.83%), which aligns with the clinical consensus for prompt and robust inflammation control in acute, extensive lesions. Simultaneously, 31.03% of patients were prescribed tetracycline-class antibiotics (often in conjunction with nicotinamide) as the initial therapeutic regimen. In comparison to prednisolone, the use of doxycycline in BP management is linked to decreased 1-year mortality, enhanced quality of life within 1 year, and a lower incidence of severe or life-threatening treatment-related adverse events, albeit resulting in less complete skin recovery at 6 weeks (106). In addition, methotrexate is also a commonly used immunosuppressant. The selection of the initial treatment regimen necessitates consideration of multiple factors. Notably, more than half (54.31%) of the patients necessitated treatment escalation, indicating that ICI-BP commonly progresses as a refractory or relapsing condition. Within this context, dupilumab has emerged as the most commonly utilized biologic agent (16, 107), effective in 13.79% of patients, highlighting its pivotal role in managing refractory ICI-BP. The effective utilization of second-line therapies such as rituximab (55, 77), omalizumab (55), intravenous immunoglobulin (75), dapsone (67), and plasma exchange (108) collectively establishes a stepped-care, multimodal treatment approach that spans from conventional immunosuppression to targeted biologics, catering to patients with diverse disease severity levels and treatment responses.

#### 4.2.2 Treatment response and disease course

The complete response rate after treatment in BP was approximately 43.10%, with a disease control rate of 92.24%. Nonetheless, the median duration from treatment commencement to disease control stood at 6 weeks, with the mean being notably elevated due to outliers. This overall indicates that managing ICI-BP typically demands several weeks, occasionally extending to a prolonged and therapy-resistant trajectory. Such gradual control could influence patients’ quality of life and potentially interrupt the consistency of their anti-cancer regimen.

#### 4.2.3 Oncologic outcomes: the dual goals of BP control and tumor control

During BP treatment, only approximately 6.03% of patients experienced documented tumor progression, while approximately 31.90% achieved tumor control by the end of follow-up. This observation is pivotal, indicating that despite the need for systemic immunosuppression due to BP, most patients effectively maintained control over the underlying malignancy. This outcome is likely due to the enduring anti-tumor immune memory prompted by ICIs and underscores clinicians’ successful management of immune-related adverse events while preserving anti-tumor efficacy. Notably, in the limited cases with recorded time from BP onset to death, the median interval was merely 3.7 months. This implies that these patients probably had advanced disease at BP onset, with their demise more likely linked to the malignancy’s severity rather than directly to BP itself.

### 4.3 Risk stratification and individualized strategy

This study, through stratified analysis, provides an in-depth revelation of the heterogeneity in ICI-BP, offering key evidence for clinical risk prediction and individualized management.

#### 4.3.1 Comparison of treatment and prognosis between mild and severe cases

This study revealed that patients with mild and severe ICI-BP did not differ significantly in demographic characteristics, oncological outcomes, or final BP remission rates. However, notable distinctions in treatment approaches were observed between the two groups, with a notably higher percentage of severe cases undergoing escalation therapy (p < 0.001). This apparently conflicting discovery underscores a fundamental principle in contemporary clinical practice: the severity grading of a disease directly influences the aggressiveness of initial and subsequent interventions. The classification of “severe” BP (e.g., extensive skin lesions, mucosal involvement, and substantial impact on daily functioning) based on CTCAE grades 3–4 inherently serves as a clear indication for systemic immunosuppressive treatment. Therefore, the observed variations in treatment are not random but rather a direct reflection of evidence-based medical decision-making. A more in-depth analysis suggests that this graded, proactive treatment approach may effectively ameliorate the potentially unfavorable prognosis in severe cases. The fact that the severe group achieved comparable final BP remission rates to the mild group in this study further bolsters this perspective. For severe cases, healthcare providers initiated prompt and aggressive administration of oral or intravenous glucocorticoids, immunosuppressants, or biologics, thereby arresting disease advancement and averting severe complications that could result from uncontrolled BP. In essence, the disparity in “treatment escalation” identified in the segmented analysis functions as a critical mediating factor that counteracts the adverse influence of “disease severity” on ultimate outcomes.

This discovery has significant clinical implications, emphasizing the importance of risk stratification by severity and stepwise treatment in managing ICI-BP. Our data illustrate the necessity and effectiveness of timely treatment escalation for patients with stage 3 ICI-BP. Moreover, our findings underscore the strengths of our study by not only detailing final outcomes but also capturing the dynamic clinical decision-making process. The “escalation decision” represents a more realistic indicator of real-world clinical practice compared to simple staging, as it integrates physicians’ comprehensive evaluation of individual patient characteristics such as symptom burden, comorbidities, and treatment response. In essence, the similarity in final outcomes between mild and severe ICI-BP patients does not suggest that disease severity is inconsequential but rather underscores the effectiveness of current active treatment approaches based on severity classification. Clinicians utilized CTCAE staging as a reference point for initiating intensive therapy, effectively improving the prognosis of severe cases to levels akin to those of mild cases. Consequently, we advocate for prompt and aggressive intervention strategies for moderate-to-severe ICI-BP.

#### 4.3.2 ICI rechallenge: a feasible strategy with risk stratification

The occurrence of BP significantly interfered with ICI treatment, leading to a discontinuation rate of 61.08% among patients in this study. This situation has prompted discussions regarding the clinical challenges associated with the resumption of ICI therapy. Our data confirm that restarting ICI treatment is generally feasible and relatively safe following effective control of BP lesions. The overall recurrence rate of BP was low at 22.2%, with most patients not experiencing recurrence after the restart, suggesting that resuming ICI therapy is a viable option for many individuals. Notably, the most common strategy employed in clinical practice was to restart the original drug (72.2%), reflecting clinicians’ tendency to prioritize the continuity of tumor treatment while considering the risk of adverse event recurrence. This observation indicates that, under effective management, “rechallenge” with the initial inducing agent is not contraindicated.

Furthermore, we identified characteristics that may be associated with a higher risk of recurrence: severe initial BP (CTCAE grade 3), mucosal involvement, and the decision to restart with the original regimen. Although the limited number of cases necessitates cautious interpretation, this trend suggests that particular caution should be exercised when considering the resumption of ICI therapy, especially the original regimen, for patients exhibiting these high-risk features. In contrast, for patients with mild (grade 2) BP without mucosal involvement, the safety of restarting therapy may be greater.

A crucial finding worthy of thorough investigation is that resuming ICI therapy did not exhibit a statistically significant correlation with the patients’ ultimate tumor outcomes. This outcome implies several layers of significance: initially, it suggests that post-BP control, resuming ICI treatment does not yield additional benefits or detriments to the ongoing tumor immunotherapy regimen; instead, the final tumor prognosis is more influenced by its inherent biological characteristics, the cumulative effectiveness of prior ICI therapy, and the synergistic impacts of other interventions. Second, this indirectly validates the safety of the decision to restart—such action does not result in a notable decline in tumor prognosis. Third, it also indicates that for certain patients (especially those with manageable BP risks necessitating aggressive tumor management), the primary advantage of resuming ICI lies in preserving the opportunity for anticancer therapy rather than necessarily altering the ultimate tumor outcome.

#### 4.3.3 Analysis of fatal cases

A detailed examination of the 11 fatal cases revealed a crucial finding: patient mortality was mainly due to the advancement of the underlying malignancy rather than the effects of BP itself. This conclusion is supported by the fact that all deceased patients were aged ≥68 years, 9/11 cases involved metastatic tumors, and the overwhelming majority (94.1%) of deceased patients had achieved disease control with BP treatment before their demise (98). Moreover, the median duration from BP diagnosis to death was merely 3.7 months, indicating that these patients often had aggressive tumors and frequently faced tumor advancement. This implies that these individuals likely had late-stage cancer at the onset of BP, and their demise was a result of the natural progression of their illness. Consequently, clinical management should prioritize effectively managing BP to enhance quality of life while ensuring that anti-tumor therapy is not compromised. For certain patients, this entails, based on risk evaluation, cautiously reintroducing or continuing ICI therapy.

## 5 Study limitations

This systematic review, based on case reports and series, has several inherent limitations. These limitations encompass publication bias, where severe or atypical cases are more likely to be reported, potential omission of a small number of relevant publications, and incomplete data reporting in the source literature. Additionally, heterogeneity among the included cases concerning tumor types, ICI regimens, and BP management strategies may impact the consistency of the results. Lastly, this study entails a correlational analysis and cannot establish causality, such as the relationship between ICI rechallenge and enhanced oncologic outcomes. The identified associations could be influenced by confounding factors; for example, patients chosen for ICI rechallenge may inherently represent a subset with superior prior treatment responses and overall health status.

## 6 Conclusion

This study comprehensively examines the clinical characteristics, treatment approaches, and impact on tumor prognosis of 116 instances of bullous pemphigoid associated with immune checkpoint inhibitors. The analysis reveals that managing ICI-related bullous pemphigoid typically involves a gradual treatment protocol tailored to the disease’s severity, commencing with glucocorticoids and advancing to biologics if needed. Although most patients can safely resume immunotherapy post-lesion remission, stringent monitoring is essential for individuals with severe initial symptoms or mucosal engagement. These results offer essential evidence-based recommendations for healthcare providers in navigating this intricate adverse event, weighing the balance between anti-tumor effectiveness and skin-related side effects, and underscoring the significance of personalized decision-making and collaborative care.

## Data Availability

Publicly available datasets were analyzed in this study. This data can be found here: The detailed database search strategies employed in this systematic review are provided in the **Supplementary Materials**. Additional data supporting the findings of this study are available from the corresponding author.
