# Supplementary material for: Immune checkpoint inhibitor-induced bullous pemphigoid: a systematic review of clinical characteristics and outcomes based on case reports
Source: Front Immunol. 2026 Mar 4;17:1745011. doi: 10.3389/fimmu.2026.1745011 (PMC12996165; doi:10.3389/fimmu.2026.1745011)
Supplement: Supplementary file 1 [file Table1.docx]

Supplementary Material

# search strategy

Pubmed

("Immune Checkpoint Inhibitors"[Mesh] OR "Programmed Cell Death 1 Receptor/antagonists and inhibitors"[Mesh] OR "CTLA-4 Antigen"[Mesh] OR "checkpoint inhibitor*"[tiab] OR "immune checkpoint inhibitor*"[tiab] OR anti-PD-1[tiab] OR anti-PD-L1[tiab] OR anti-CTLA-4[tiab] OR "PD 1"[tiab] OR "PD1"[tiab] OR "PD-1"[tiab] OR "PD L1"[tiab] OR "PDL1"[tiab] OR "PD-L1"[tiab] OR nivolumab[tiab] OR pembrolizumab[tiab] OR cemiplimab[tiab] OR dostarlimab[tiab] OR ipilimumab[tiab] OR atezolizumab[tiab] OR durvalumab[tiab] OR avelumab[tiab]) AND ( "Pemphigoid, Bullous"[Mesh] OR "bullous pemphigoid"[tiab] OR BP[tiab]) AND ("Case Reports"[Publication Type] OR "Case Reports"[tiab] OR "case series"[tiab] OR "case of"[tiab])

WoSCC

TS=("checkpoint inhibitor*" OR "immune checkpoint inhibitor*" OR "anti-PD-1" OR "anti-PD-L1" OR "anti-CTLA-4" OR nivolumab OR pembrolizumab OR cemiplimab OR dostarlimab OR ipilimumab OR atezolizumab OR durvalumab OR avelumab) AND TS=("bullous pemphigoid" OR BP) AND TS=("case report" OR "case series" OR "case of")

Embase

| #1 | 'immune checkpoint inhibitor'/exp OR 'immune checkpoint inhibitor' OR 'programmed death 1 receptor'/exp OR 'programmed death 1 receptor' OR 'cytotoxic t lymphocyte antigen 4'/exp OR 'cytotoxic t lymphocyte antigen 4' |
| --- | --- |
| #2 | 'checkpoint inhibitor*':ab,ti OR 'immune checkpoint inhibitor*':ab,ti OR 'anti pd 1':ab,ti OR 'anti pd l1':ab,ti OR 'anti ctla 4':ab,ti OR nivolumab:ab,ti OR pembrolizumab:ab,ti OR cemiplimab:ab,ti OR dostarlimab:ab,ti OR ipilimumab:ab,ti OR atezolizumab:ab,ti OR durvalumab:ab,ti OR avelumab:ab,ti |
| #3 | #1 OR #2 |
| #4 | 'bullous pemphigoid'/exp OR 'bullous pemphigoid' |
| #5 | 'bullous pemphigoid':ab,ti OR bp:ab,ti |
| #6 | #4 OR #5 |
| #7 | 'case report':ab,ti OR 'case series':ab,ti OR ‘case of’:ab,ti |
| #8 | #3 AND #6 AND #7 |
